# Benchmarking Sample Preservation Strategies for Clinical Single-Cell Proteomics

**DOI:** 10.1016/j.mcpro.2026.101676

**Published:** 2026-10-06

**Authors:** Heloísa Monteiro do Amaral-Prado, Jackson Gabriel Miyamoto, Daniella de Figueiredo, Elaine Cristina Cardoso, Fábio Malta de Sá Patroni, Romênia Ramos Domingues, Bianca Alves Pauletti, Adriana Franco Paes Leme

**Affiliations:** Laboratório Nacional de Biociências (LNBio), Centro Nacional de Pesquisa em Energia e Materiais (CNPEM), Campinas, São Paulo, Brazil

**Keywords:** clinical samples, mass spectrometry, cellenONE, Orbitrap Astral, peripheral blood mononuclear cells, sample preservation, cryopreservation, fixation

## Abstract

Single-cell proteomics (SCP) enables direct characterization of cellular states at proteome resolution but remains limited in clinical studies due to challenges in sample collection, storage, and transportation. Here, we systematically evaluated preservation strategies for SCP by comparing fresh, cryopreserved, and paraformaldehyde-fixed cells using HeLa cells as a benchmark model (n = 159 cells). Both strategies maintained comparable proteome coverage, but fixation altered protein abundance, whereas cryopreservation largely preserved the proteomic profile. To assess the translational applicability of this workflow, we analyzed single peripheral blood mononuclear cells from one healthy donor, comparing freshly isolated cells (n = 77) with cells subjected to cryopreservation followed by extended-term storage (n = 75). In peripheral blood mononuclear cells, cryopreservation did not significantly affect protein group identifications, cellular morphology, proteome coverage, or global proteomic landscape. Together, these results demonstrate that cryopreservation provides a feasible strategy for preserving primary immune cells for downstream SCP under the conditions evaluated here, while comparative preservation strategies using single HeLa cells highlight the advantages and challenges of fixation and cryopreservation for future clinical applications.

## Highlights

• Both sample preservation strategies (fixation and cryopreservation) showed similar proteome coverage and variation for single HeLa cells.
• The association between cellular volume and cell-cycle protein abundance was preserved in fixed and cryopreserved single HeLa cells despite storage-induced morphological changes.
• Cryopreserved single HeLa cells showed a proteome profile similar to fresh controls, whereas fixation reduced metabolic proteins.
• Up to 3500 and 1500 protein groups were quantified in single HeLa cells and PBMCs, respectively.
• SCP enables the identification of major immune-cell subpopulations in cryopreserved PBMCs.

## Graphical Abstract

**Figure:**
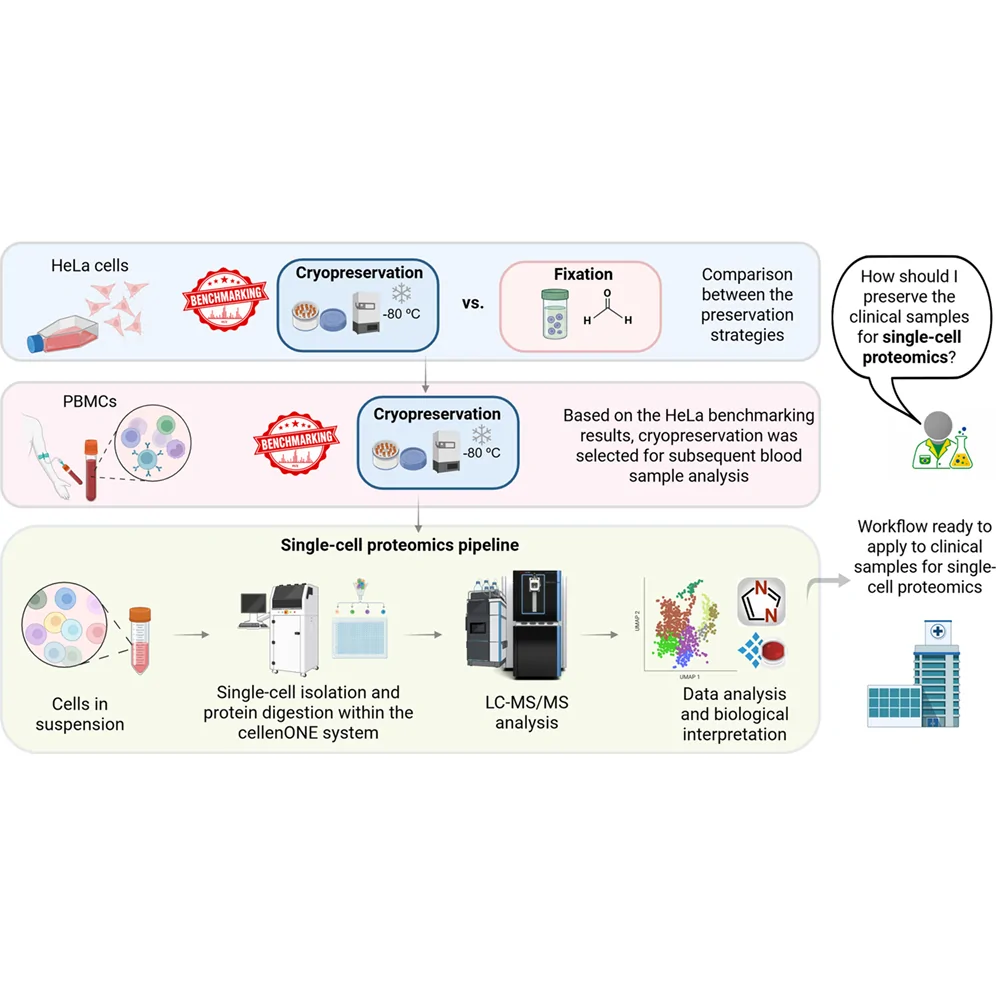

## In Brief

Single-cell proteomics studies using clinical samples remain limited. Clinical workflows often require sample storage, making preservation strategies critical. In this study, we assess selected practical approaches, including cryopreservation and fixation, first using HeLa cells as a controlled model, and subsequently evaluating cryopreservation in PBMCs as a clinically relevant sample. Importantly, this study was not intended to exhaustively evaluate all preservation strategies, but to provide practical insights for future clinical SCP studies.

Single-cell proteomics based on mass spectrometry (SCP) has emerged as an analytical method in the scientific field over the last decade, and its use is growing rapidly 1, 2, 3, 4, 5, 6. Because proteins are more closely related to phenotype than RNA, SCP promises to advance cell biology by revealing cellular mechanisms that cannot be elucidated by single-cell RNA sequencing 7, 8, 9. Importantly, SCP can also uncover new potential disease signatures in cell subtypes. Nevertheless, applying SCP to complex and heterogeneous cell populations, such as those found in blood, remains challenging 10, 11.

Blood provides a readily accessible source of biological information and reflects systemic alterations associated with disease progression 12, 13, 14, 15. Although circulating immune cells represent only a small fraction of blood cells, they play essential roles in tumor surveillance, immune regulation, and metastatic dissemination. Therefore, applying SCP to blood-derived immune cells offers a powerful approach to resolve immune heterogeneity and identify functional cellular states associated with cancer progression (16).

One major challenge of working with clinical samples at the SCP level is the need for a multidisciplinary, synchronized, and trained team, as well as defined protocols for cell collection and storage. Additionally, the sample shipment depends on the region where the sample was collected. Shipment may therefore take several days, and if samples are stored using dry ice or liquid nitrogen, these must be replaced during the shipment period. Efforts to evaluate cell shipment for SCP under cryopreservation with liquid nitrogen (−196 °C) (17) or paraformaldehyde (PFA) fixation 18, 19, 20 have already been evaluated by several groups using immortalized cells and tissue from an *in vivo* mouse model. However, these studies did not apply the storage conditions to preserved clinical Human biofluid samples for extended (days or a few weeks) or long-term (months or years).

PFA fixation and cryopreservation are widely used strategies for preserving clinical samples before downstream analyses. PFA fixation facilitates extended-term storage and sample handling but may alter proteome composition through protein crosslinking, potentially affecting protein extraction efficiency and downstream molecular measurements (21). In parallel, cryopreservation, typically performed with fetal bovine serum (FBS) and dimethyl sulfoxide (DMSO), preserves cell viability and enables long-term biobanking; however, its effects on single-cell proteomic profiles remain insufficiently characterized (22). Therefore, several factors, including exogenous protein contamination, cryoprotectant toxicity, morphological alterations, and freezing-induced proteomic changes, have the potential to influence protein quantification and impact downstream biological interpretation. Establishing preservation strategies that preserve proteome integrity is thus critical for the reliable application of single-cell proteomics to clinical samples.

Herein, single HeLa cells were cryopreserved at −80 °C or fixed with 2% PFA and stored at 4 °C. The cells were then stored for 1 day (short-term) or 8 days (extended-term) before being isolated on the same 384-well plate to facilitate an accurate comparison of different storage conditions. This process was then applied to freshly isolated and cryopreserved peripheral blood mononuclear cells (PBMCs) from one healthy individual following extended-term storage to validate a clinically compatible SCP workflow. It is important to note that this study was not intended to exhaustively evaluate all possible strategies for handling cells collected in clinical settings, but rather to assess selected, practical preservation approaches and demonstrate their applicability to clinical samples, with potential future value in clinical studies.

## Experimental Procedures

### Ethical Approvals

Blood sample was collected from one healthy individual. The study was approved by the Ethics Committee of the Piracicaba Dental School (FOP), São Paulo, SP, Brazil, through protocol CAAE: 54995522.0.0000.5418. All procedures involving human participants were conducted in accordance with the principles of the Declaration of Helsinki.

### Cell Culture of HeLa Cells

HeLa cells (CCL-2) were purchased from the Rio de Janeiro Cell Collection (Banco de Células do Rio de Janeiro, Brazil) and were authenticated by the ATCC. The cells were maintained in Dulbecco’s modified Eagle medium (DMEM) supplemented with 10% FBS and 1% antibiotics (penicillin and streptomycin) at 37 °C and 5% CO2. For single-cell proteomics (SCP) analysis, cells were cultured in medium-sized flasks (T75) and collected at 80%–90% confluence. Next, cells were trypsinized and washed twice with 1 × phosphate-buffered saline (PBS).

### Cryopreservation and Fixation of HeLa Cells for SCP

HeLa cells (1 × 10^6^ cells/ml) were cryopreserved using a freezing medium composed of 95% FBS and 5% DMSO. Cells were rapidly transferred to a freezing container (Mr Frosty, Sigma, #C1562) for 16 h and stored in an ultra-freezer (−80 °C). After the cryopreservation period, cells were rapidly thawed in a dry bath at 37 °C, washed twice with PBS to remove the freezing media, and stained for viability prior to single-cell isolation. The fixation was performed using 1 ml of a PFA solution (ChemCruz, #sc-281692) diluted to a final concentration of 2% with PBS and incubated for 15 or 30 min, followed by two washes with 1 ml of PBS to remove the fixation solution.

### Peripheral Blood Mononuclear Cell Extraction and Cryopreservation for SCP

The collected peripheral blood was transported at room temperature from the location of the collection to the laboratory where it was quickly processed to extract and cryopreserve the PBMCs for subsequent SCP experiments. Fresh PBMCs were used as the experimental control for SCP. Cells were extracted from 16 ml of peripherical blood in EDTA tubes from the healthy individual, diluted in PBS at a 1:1 proportion, and carefully added to the same volume of Ficoll-Paque Premium density gradient media (Cytiva, #GE17-5442-02) without mixing. Then, the solution was centrifuged at 600*g* for 30 min, and PBMCs were collected, followed by two washes with PBS and centrifugation at 200*g* for 5 min.

Following the extraction, 1 × 10^7^ cells/ml of PBMCs were cryopreserved using a freezing medium composed of 90% FBS and 10% DMSO. Cells were rapidly transferred to a freezing container (Mr Frosty, Sigma, #C1562) for 16 h and stored in an ultra-freezer (−80 °C). After the cryopreservation period, cells were rapidly thawed in a dry bath at 37 °C, washed twice with PBS to remove the freezing media.

### Cell Viability Staining before Single-Cell Isolation

Before single-cell isolations, cells were incubated with a Zombie NIR fixable viability kit (BioLegend, #423105) at a final concentration of 1:250 for 15 min at room temperature. For fixed-cell samples, viability staining was performed before the fixation step to allow the distinction of viable and non-viable cells based on plasma membrane integrity before fixation.

### Detection and Isolation of Cells Assisted by the cellenONE X1 Robot

Cell isolation and sample preparation for SCP were performed using a cellenONE X1 robot (Cellenion, Lyon, France). All solutions were prepared using MS-grade reagents, degassed before use, and loaded into a medium-sized uncoated piezoelectric dispensing capillary (PDC).

After cell viability staining, the cells were washed twice with PBS and resuspended in previously filtered (Millipore Steritop vacuum bottle top filter, Merck, #SCGPS02RE) PBS at a final concentration of 200 to 300 cells/μl. A master mix, consisting of 100 mM triethylammonium bicarbonate buffer, 0.05% n-dodecyl-β-D-maltoside (Sigma, #D4641) and 3 ng/μl trypsin (Waters, #186010108) in de-gassed MS-grade water, was dispensed into each well of a clean Eppendorf LoBind 384-well PCR plate (Merck, #EP0030627300). For the SCP experiments with HeLa cells stored under different conditions, 1% DMSO (Sigma, #472301) and 0.01% trypsin enhancer (ProteaseMAX, #V2072) were included in the master mix. The transmission channel (T) was initially used for cell selection, followed by fluorescence channel (F) analysis using a red LED for the negative selection of viable cells (T > F). To ensure analysis of all cell particles, the detection parameters were set to a wide range: diameter of 3 to 100 μm and elongation up to 4. The following cell isolation parameters were optimized for each cell population: HeLa cells (n = 20 for each storage condition), diameter of 20 to 40 μm and elongation up to 1.8; PBMCs, diameter of 9 to 30 μm and elongation up to 1.8. During the cell isolation, the 384-well plate was maintained at 10 °C and 45% humidity. Next, the plate was incubated at 50 °C for 2 h. The humidity was maintained at 85% to minimize evaporation, and wells were hydrated with 500 nl of water. In the second round of hydration, 500 nl of 3 ng/μl trypsin resuspended in degassed MS-grade water was used, as described by Matzinger *et al.* (23) and Bubis *et al.* (24). After 2 h of incubation, 4 μl of 0.1% formic acid (FA) was added to each well using an electronic repeater pipette to quench the trypsin activity. The plate was then dried in a Thermo SC250EXP SpeedVac concentrator at room temperature and stored at −20 °C until injection. Wells containing multiple cells (n = 10 cells; denominated as a library) were processed through the same workflow to construct sample-size-comparable spectral libraries.

### Quality Controls Used for SCP Experiments

As experimental controls for SCP during cell isolation, we included blank wells on the cell isolation plates that contained only the master mix without any cells. An additional cleaning step of the PDC (referred to as “sterilization”) was performed before and after cell isolation, with three solutions used in sequence (0.5% sodium hypochlorite, 3% hydrogen peroxide, and 70% ethanol), followed by a “sci-clean” task and two flushes of the PDC with 250 nl of MS-grade water at a wash station. Before proteomic analysis, images of the cells isolated in the PDC corresponding to each well were manually inspected, and those containing more than one isolated cell or contaminated by debris were excluded from subsequent analysis.

### LC-MS/MS Analysis

Samples in the 384-well plates were resuspended in 4.0 μl of 0.1% FA, and 3.5 μl was analyzed using a Vanquish Neo UHPLC chromatographic system coupled with an Orbitrap Astral mass spectrometer (Thermo Fisher Scientific, Germany). The chromatographic separation of peptides was performed on a C18 Aurora Ultimate TS column (25 cm × 75 μm ID, 1.7 μm). The column oven (IonOpticks) temperature was 50 °C. The system maximum pressure was 1200 bar with a loading volume of 1 μl, with fast loading and wash options enabled. A gradient elution was conducted at a throughput of 50 samples per day using an active gradient. The percentage of buffer B (80% ACN in water with 0.1% FA) initially increased from 0% to 40% over 19.5 min at a nominal flow rate of 200 to 450 nl/min, followed by a gradual increase to 99% buffer B from 19.5 to 22 min at a flow rate of 300 nl/min, which was maintained for an additional 5 min.

Single-cell-derived peptides were acquired in positive mode using the FAIMS Pro interface, with a compensation voltage set to −48 V and a spray voltage of 1800 V. Orbitrap MS1 spectra were acquired at a resolution of 240,000, with a scan range of 400 to 800 m/z, a normalized automatic gain control target of 500%, and a maximum injection time of 100 ms. Data-independent acquisition (DIA) of MS2 spectra was performed on the Astral mass spectrometer using the same scan range, an automatic gain control of 800%, and loop control set to 0.6 s per cycle. The DIA method consisted of 20 consecutive MS/MS scans using 20 m/z-wide isolation windows, with a cycle time of 0.6 s. The injection time was set to 60 ms for HeLa samples and 80 ms for PBMC samples. Precursor ion fragmentation was achieved using higher-energy collisional dissociation with a normalized collision energy of 27%. Data acquisition was performed for 24 min once elution began. Following data collection, the FAIMS voltage was set to −70 V during the washing and equilibration steps to minimize contaminant transmission to the mass spectrometer.

### Data Processing and Protein Identification

For Spectronaut DIA searches (versions 19.9 and 20.1), raw files were converted to.htrms files using the HTRMS converter. Protein identification was carried out against the UniProt human reference proteome (UP000005640, 20,654 entries, downloaded in January 2025), supplemented with the common Repository of Adventitious Proteins (cRAP; 116 entries) and common Repository of FBS Proteins (199 entries) (25) to eliminate common mass spectrometry contaminants and proteins derived from FBS, respectively. For DIA-NN (version 2.3), an *in silico* spectral library was created using the same human proteome FASTA file used in Spectronaut, with the “contaminant” option selected. Protein groups (PGs) from the contaminant repositories were removed. Blank samples were independently analyzed by directDIA+ for each condition as sample replicates in Spectronaut and with MBR enabled in DIA-NN. The detailed parameters used for Spectronaut and DIA-NN software are described in Data S2 and S3, respectively.

### Data Filtering and Integration with cellenONE Morphometric Information

PG tables generated by Spectronaut and DIA-NN (.pg_matrix.tsv) were first filtered to remove contaminants. Missing values were replaced with zeros. Proteins found in the blank controls were excluded, and cells with less than a 1.35-fold change based on the blank average were removed from subsequent analyses. The morphology data (diameter, elongation, and circularity) were obtained using cellenONE software and integrated with the proteomic tables by remapping the row 1, 2, 3, 4, 5, 6, 7, 8, 9, 10, 11, 12, 13, 14, 15, 16, 17, 18, 19, 20, 21, 22, 23, 24 and column 1, 2, 3, 4, 5, 6, 7, 8, 9, 10, 11, 12, 13, 14, 15, 16 information provided by cellenONE to the well positions in the 384-well plates, with rows A–P and columns 1 to 24.

Cell volume (μm^3^) of HeLa cells was estimated from the measured cell diameter and elongation parameters. The elongation factor formula was retrieved by cellenONE software as the ratio between the maximum Feret diameter and the short side of the equivalent rectangle (ε = D/Rf, Data S2). Then, the estimated cell volume was calculated according to the following formula:

$$Volume\;\left(\mu m^{3}\right)=\pi D^{3}/6\sqrt{\varepsilon }$$

### Experimental Design and Statistical Rationale

This study was designed to evaluate the impact of preservation strategies on single-cell proteomic measurements and to establish a workflow compatible with clinically relevant samples. Consequently, PBMC experiments were performed using a single healthy donor to minimize biological heterogeneity while enabling direct comparison between preservation conditions. Because the PBMC comparison was performed using cells from a single donor, statistical comparisons were interpreted as within-donor experimental comparisons and were not intended to support population-level inference.

HeLa cells were used as a benchmark model to compare freshly processed cells, PFA-fixed cells, cells stored at 4 °C, and cryopreserved cells after short-term (1 day) and extended-term (8 days) storage. A total of 160 single HeLa cells were initially isolated across the experimental conditions; however, one cell was excluded (condition: −80 °C (1 day)) because the corresponding well showed evidence of contamination, resulting in 159 cells retained for downstream SCP analysis. To validate the selected preservation strategy in primary human cells, 152 single PBMCs obtained from one healthy donor were analyzed, including 77 freshly isolated and 75 cryopreserved cells.

Freshly processed cells were used as reference controls for all preservation conditions. Blank wells containing no cells were included throughout sample preparation and LC-MS/MS acquisition to monitor background contamination. Cell morphology (diameter, elongation, and circularity) was measured prior to isolation using the cellenONE imaging system and used as an additional quality-control metric. No technical replicate LC-MS/MS injections were acquired because each single-cell preparation constituted a unique analytical measurement, consistent with current SCP workflows.

Cells failing automated image-based quality control or containing insufficient proteomic information following database searching were excluded prior to downstream analyses according to predefined quality control criteria. The same filtering criteria were applied across all experimental groups.

Finally, statistical analyses were performed in R (v4.4.1; https://www.r-project.org/) and Python (v3.11; https://www.python.org/). Data distributions were assessed prior to statistical testing. Comparisons between two groups were performed using two-sided Wilcoxon rank-sum tests or Welch’s t-tests, depending on data distribution. Multiple-group comparisons were performed using one-way ANOVA followed by Tukey's post hoc test when assumptions of normality and homogeneity of variance were satisfied. Otherwise, the Kruskal-Wallis test followed by Dunn’s post hoc test was used. Differential protein abundance analysis was performed using Benjamini–Hochberg correction for multiple testing. Adjusted *p*-values <0.05 were considered statistically significant. Functional enrichment analyses were performed using g:Profiler with g:SCS multiple-testing correction.

## Post-Analysis

### Metrics

Bar charts displaying the average and SD of the PGs and peptides were generated using the *sns.barplot* function in the Seaborn package (v0.13.2) (26). The CV was evaluated for each PGs across cells within each experimental condition as the percentage of the ratio of the SD to the mean. Missing values (NA) were excluded from the calculation. Infinite values resulting from division by zero were treated as missing values. The percentage of peptides with 0, 1, or ≥2 missed cleavages was calculated from *Stripped.Sequence* column for each sample and summarized across replicates for each condition.

Data normality was evaluated using the Shapiro-Wilk test. Pearson’s correlation was applied to normally distributed data, and Spearman’s correlation was used otherwise. Positive and negative correlations were indicated by ρ > 0 and ρ < 0, respectively. Statistical significance was defined as *p* < 0.05. Finally, Venn diagrams were generated using the web tool InteractiVenn (25).

### Dimensionality Reduction

Dimensionality reduction was performed using principal component analysis (PCA) and uniform manifold approximation and projection (UMAP) implemented in the Scanpy package (27). MS1 intensity values were transformed into a log format using a natural logarithm with a pseudocount of 1 (log1p). PCA was then performed on the log-transformed data without variance scaling. Next, UMAP plots were generated from a k-nearest neighbor graph constructed using the first 20 principal components with Leiden clustering (28) across a range of resolution parameters (0.1-3). The optimal resolution was selected based on biological interpretability and marker expression patterns. Specifically, for HeLa cells, a resolution of 0.2 was selected because it yielded two major clusters corresponding predominantly to fixed and unfixed cells, whereas for PBMCs, a resolution of 1 was selected because it yielded six clusters with marker profiles corresponding to major immune-cell populations.

To assess whether cellular volume contributed to the structure of the single-cell proteomic profiles of HeLa cells, UMAP embeddings were generated before and after regression of the estimated cell volume. For the volume-adjusted analysis, estimated cell volume was regressed out from the proteomic data using *scanpy*.*pp*.*regress_out*(), followed by feature scaling with a maximum absolute value of 10. PCA, neighborhood graph construction, and UMAP embedding were subsequently performed using the same parameters as for the unadjusted analysis.

Differential expression analysis was performed using the Wilcoxon rank-sum test implemented by the *sc*.*tl*.*rank_genes_groups*() function, in which cells in a Leiden cluster were compared with all other cells. Cluster markers were filtered using the following criteria: adjusted *p* < 0.05, log fold change >1, expression in >60% of cells within the cluster (pct_in), and expression in <20% of cells outside the cluster (pct_out). Dot plots were generated to visualize the expression patterns of the abundant protein markers across clusters with per-protein standard scaling enabled (*standard_scale =* “*var*”). The filtered results were exported as tab-delimited files for downstream analyses.

### Correlation of Cellular Volume and Cell-Cycle Protein Analysis in Single HeLa Cells

The correlation between single-cell HeLa volume and cell-cycle-related protein abundance was investigated by analyzing the abundance of proteins involved in G1/S transition, DNA replication, G2/M transition, and chromosome segregation. In total, 31 proteins were analyzed: CDK2, CDK4, CDK6, PCNA, MCM2, MCM3, MCM4, MCM5, MCM6, MCM7, RPA1, RPA2, RPA3, CCNA2, CCNB1, CCNB2, CDK1, PLK1, AURKA, AURKB, BUB1B, MAD2L1, CDC20, CENPF, SMC1A, SMC3, STAG1, STAG2, TOP2A, KIF11, and KIF20A. To reduce the influence of highly sparse comparisons and obtain more reliable correlation estimates, the analyses were restricted to proteins with at least 5 cells with measurements for both cellular volume and protein abundance. *P*-values from the multiple correlation tests were adjusted for multiple comparisons using the Benjamini–Hochberg method. Finally, the abundance of each detected cell-cycle protein was standardized using z-score transformation, and the mean z-score across the cell-cycle protein panel was calculated for each cell to generate a cell-cycle protein score. The association between estimated cell volume and the cell-cycle protein score was then assessed separately for each experimental condition using Spearman’s rank correlation (ρ).

### Volcano Plots

The terminology for conditions was standardized, and experimental controls were used as references. For each condition of stored HeLa cells or PBMCs, differential abundance was assessed using Welch’s *t* test against the control (Fresh conditions). Zero values in the protein abundance matrix were treated as NA and excluded from statistical testing. For fold-change analysis, a pseudocount of one was added to the mean abundance of each protein in the comparison and control groups before calculating log2 fold changes. Proteins were tested when at least two non-missing measurements were available in each group. Proteins with |log_2_ fold change| ≥ 1 and adjusted *p* < 0.05 were considered significantly regulated.

### Gene Ontology Annotation

Gene Ontology (GO) enrichment analysis was performed using the g:Profiler Python interface (gprofiler) 29, 30 to identify enriched biological processes and cellular components among differentially abundant proteins in each experimental condition of HeLa cells and PBMCs under storage conditions. Analyses were restricted to *Homo sapiens* and the GO Biological Process (GO:BP) category. Enriched terms were retained at a significance threshold of adjusted *p*-value <0.05 after g:SCS multiple testing correction. Enrichment significance was expressed as −log10-transformed adjusted *p*-values. For each condition, the top 10 enriched GO terms ranked by adjusted *p*-value were selected. The number of overlapping genes contributing to each term was obtained from the reported intersection size. Dot plots were generated using Seaborn, where dot size represents gene overlap and dot color represents enrichment significance (−log10 adjusted *p*-value).

### Immune Cell Population Annotation Based on the Human Gene Atlas

The cell type annotations were determined based on a search of the cluster markers against the BioGPS database available as a “Human Gene Atlas” option in the Enrichr web tool (31), which contains information on upregulated genes in human tissue.

## Results

### Cryopreserved and Fixed HeLa Cells Showed Similar Proteome Coverage, but Cryopreserved Cells Exhibited a Proteomic Profile More Similar to Fresh Controls

In the initial SCP experiments, HeLa cells were cultured and compared using different conditions: 1) freshly isolated on the day of the experiment (37 °C; control); 2) fixed in 2% PFA and stored at 4 °C (fixation time of either 15 or 30 min); or 3) cryopreserved at −80 °C. For conditions 2, 3, the time of storage was either 1 day (short-term storage) or 8 days (extended-term storage) (Fig. 1). A fourth condition was added as a negative control for the storage condition, in which cells were stored for both short- and extended-term at 4 °C without any additional treatment. The selected storage period of 8 days reflects a practical time window for sample shipment and processing, and 2% PFA was selected because it is a concentration widely used for cell fixation in flow cytometry, while providing robust cellular preservation, and has been shown to maintain broadly comparable proteomic performance to lower PFA concentrations in recent SCP studies 18, 19. Importantly, all the different samples were isolated on the same 384-well plate within the cellenONE X1 system to minimize technical artifacts, such as batch effects, and proteins found in the blank controls (master mix without an isolated cell) were excluded from the analysis.

**Fig. 1:**
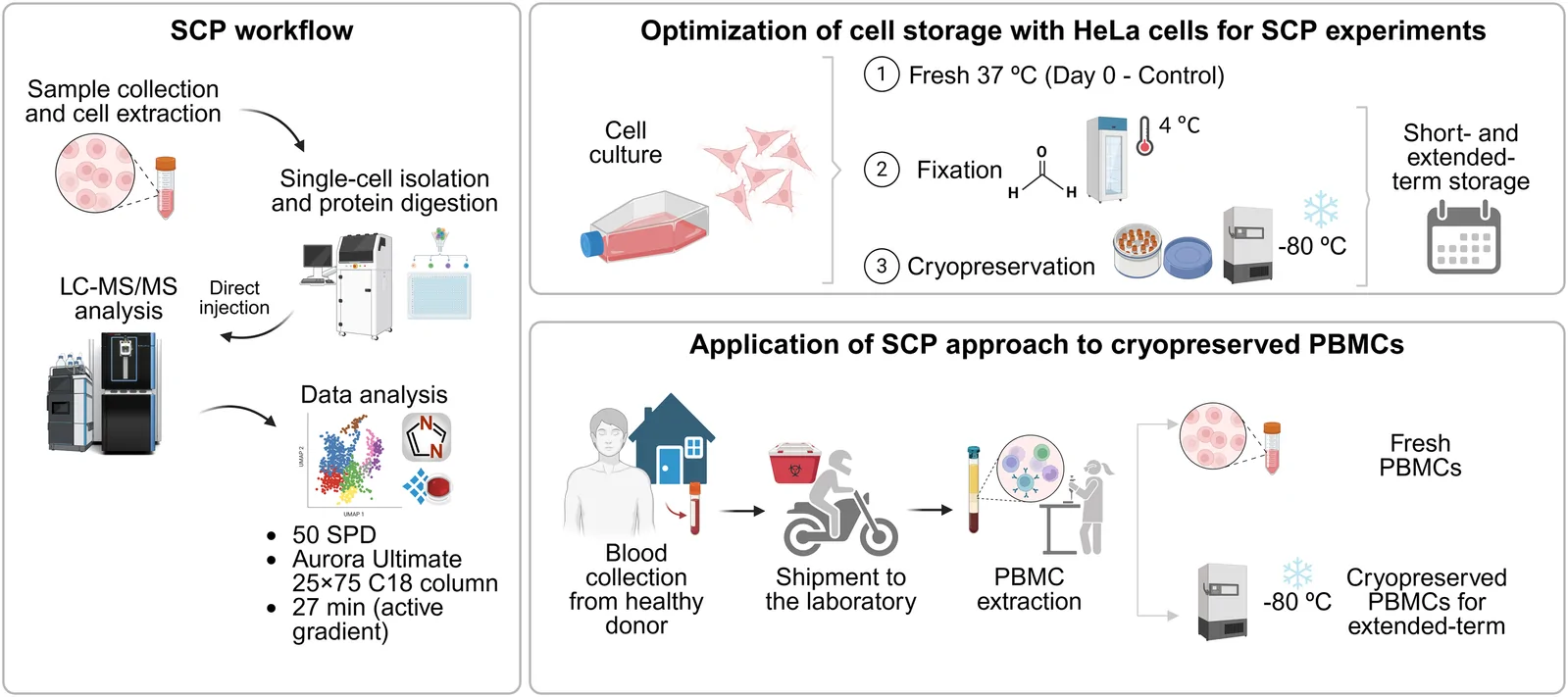
**Experimental workflow for benchmarking preservation strategies and application to clinical samples**.

We first evaluated the performance of different DIA proteomic search tools for protein identification in HeLa cells. DIA-NN (version 2.3) software (32) consistently yielded a higher number and diversity of PGs compared to Spectronaut (versions 19.9 and 20.1), mainly due to a greater number of unique identifications in the biological samples (Fig. 2*A* and Supplemental Tables S1 and S2). Notably, DIA-NN also produced more stringent results for the blank controls, which avoided inflating protein identifications due to background signals (Fig. 2*A* and Supplemental Table S3). DIA-NN showed improved sensitivity in biological samples 32, 33 while producing fewer identifications in blank controls, indicating better distinction of sample-derived signals from background noise. Therefore, we selected DIA-NN for downstream SCP analyses in this study.

**Fig. 2:**
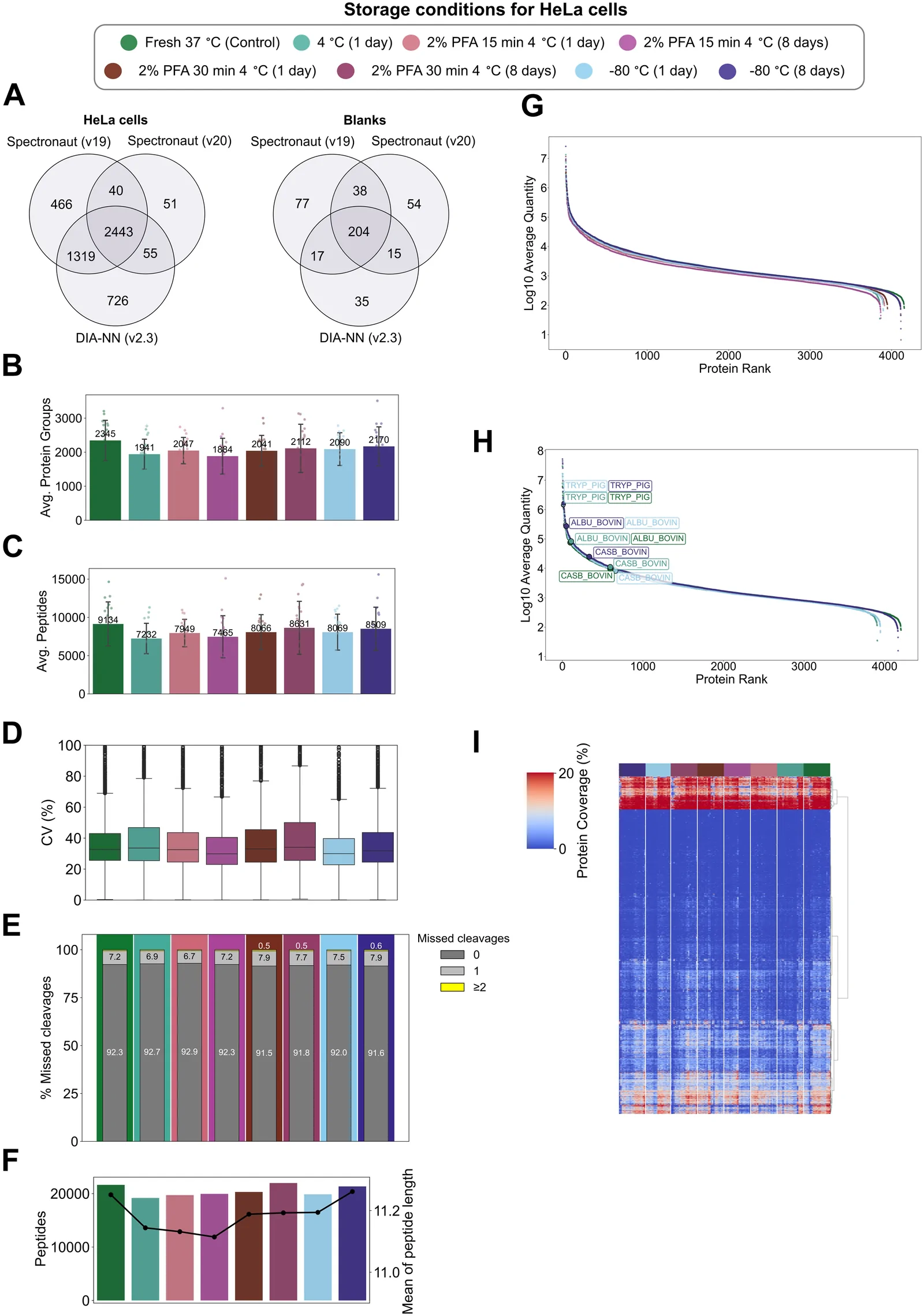
**Effects of storage conditions on global proteomic metrics in single HeLa cells.***A*, overlap of proteins identified in single HeLa cells (n = 20 single HeLa cells per condition, except for −80 °C for 1 day, for which n = 19) and blanks (n = 6) across different search engines. Average ± SD of PGs (*B*) and peptides (*C*) according to storage conditions. *D*, CV within the tested conditions. *E*, percentage distribution of peptides according to the number of missed cleavages: 0, 1, or ≥2. *F*, absolute number (*bars*) and length distribution (*lines*) of identified peptides according to storage conditions. *G*, dynamic range of protein abundances. *H*, impact of cryopreservation on protein abundance profiles. Proteins related to sample processing and cryopreservation are highlighted for illustrative purposes. *I*, heatmap of protein sequence coverage percentage. The heatmap color scale was truncated at the 95 th percentile of the distribution to prevent compression of observations. Coverage values exceeding the cap are saturated at the upper color limit. PG, protein group.

Importantly, using the same false discovery rate threshold of 1% enabled a more direct comparison of PG and peptide identification performance between the two search tools. However, these analyses do not represent an exhaustive comparison between DIA-NN and Spectronaut. A comprehensive benchmarking of the two software tools was beyond the scope of the present study, and therefore the observed differences should not be interpreted as a systematic assessment of their relative performance. These results are instead intended to provide context for the software selection used in the present workflow.

An average of 2345 PGs (Figs. 2*B*) and 9134 peptides (Fig. 2*C*) were observed in control samples (fresh, 37 °C), with up to 3510 PGs identified in a single HeLa cell. The average number of PGs decreased in cells subjected to cryopreservation or fixation, with both strategies yielding similar numbers of PGs and peptides after short- and extended-term storage. Cells stored at 4 °C for 1 day without fixation or cryopreservation exhibited the lowest number of protein identifications. After 8 days of storage at 4 °C, 59% of cells were dead, rendering single-cell isolation infeasible (Supplemental Fig. S1). Consequently, this condition was excluded from further analysis.

The impact of storage conditions on proteome variability and digestion efficiency was next assessed. The median CV (Fig. 2*D*) and percentage of missed cleavages (Fig. 2*E*) remained consistent across the conditions, with the lowest variations in fresh cells. Moreover, the shared stripped sequence length (Fig. 2*F*) was similar across conditions, supporting the consistency of the proteome representation, as well as the dynamic range, spanning approximately five orders of magnitude (Fig. 2*G*).

To evaluate the effect of a high FBS concentration during cryopreservation 17, 34, fresh and cryopreserved samples were compared (Fig. 2*H*). Although the albumin abundance increased under both cryopreserved conditions (log_2_ fold change 2–2.2) and alpha and beta casein showed higher levels after 8 days of cryopreservation (log_2_ fold change 1.3–2.2), the overall quantitative profiles were comparable to those of fresh cells (Fig. 2*H*). Furthermore, protein sequence coverage (Fig. 2*I*) was similar across conditions, supporting the consistency of the proteome representation.

Next, PCA (Fig. 3*A*) and UMAP results (Fig. 3, *B–D* and Supplemental Table S4) revealed a clear separation among cells treated with PFA compared to other conditions. Two clusters were retrieved using the Leiden algorithm (cluster 0, predominance of fixed cells and cluster 1, predominance of unfixed cells), with the identified markers mainly related to lumen cellular compartments and metabolic molecular functions (Supplemental Table S5).

**Fig. 3:**
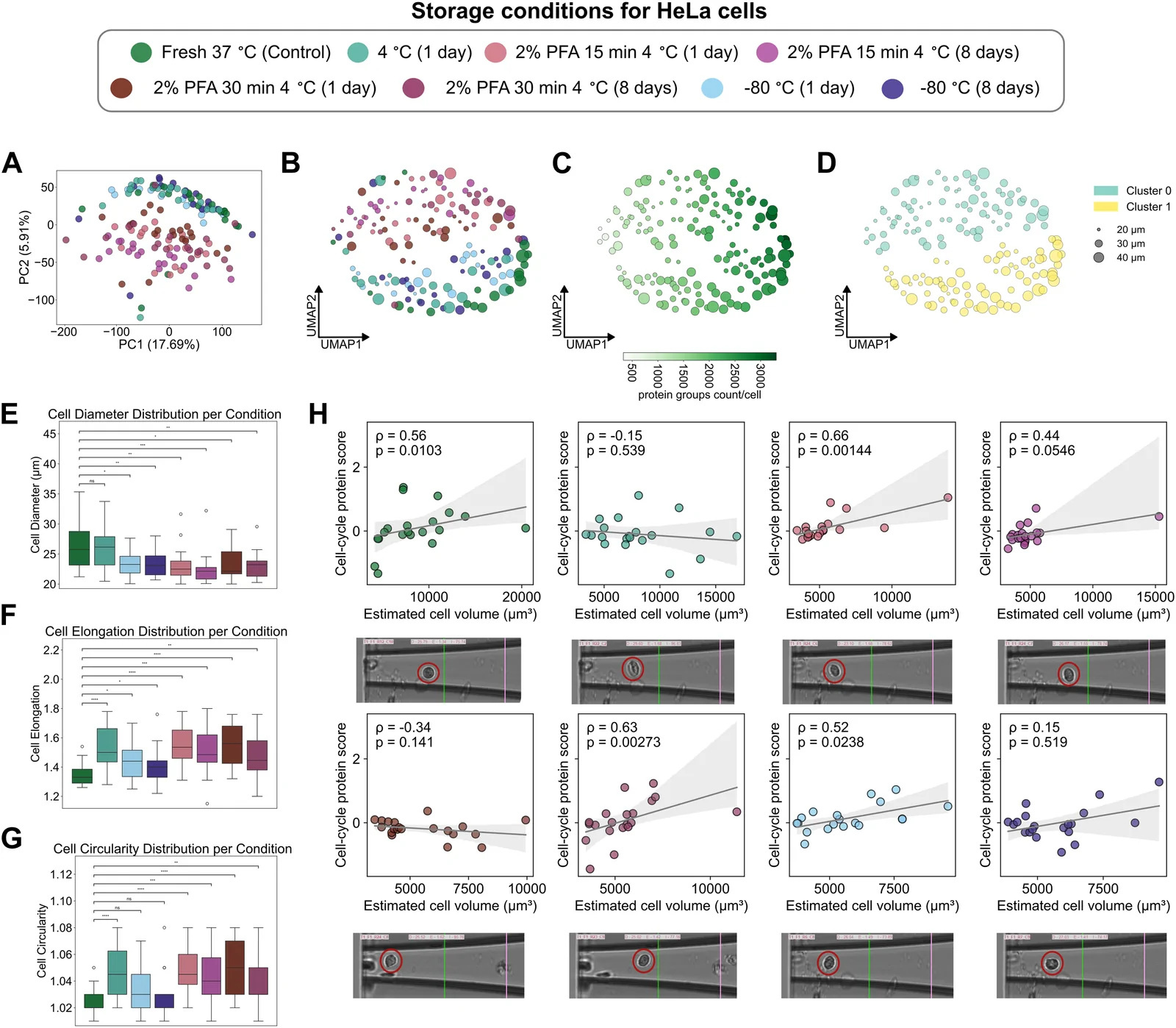
**Morphological and proteomic effects of cryopreservation and fixation in single HeLa cells.** PCA (*A*) and UMAP plots (*B*–*D*) were based on protein quantities. Each *dot* represents one cell, and *colors* indicate the storage conditions (*B*), the number of PGs identified per cell (*C*), and Leiden clusters (resolution of 0.2) (*D*). The *dot size* in the UMAP plots corresponds to the cell diameter (range for isolated cells: 20–40 μm). *E*–*G*, *boxplots* showing single-cell diameter (*E*), elongation (*F*), and circularity (*G*) distributions by storage conditions. Mann–Whitney U test with FDR correction was used to determine the statistical differences between the groups; Significance levels are indicated as: ns (not significant), 0.05 < *p* ≤ 1.00; ∗, 0.01 < *p* ≤ 0.05; ∗∗, 0.001 < *p* ≤ 0.01; ∗∗∗, 0.0001 < *p* ≤ 0.001; ∗∗∗∗, *p* ≤ 0.0001. *H*, correlation between estimated cell volume (μm^3^) and cell-cycle protein score. Cell-cycle-related proteins were selected from the quantified PGs, and their abundance values were extracted for each cell. To account for differences in protein abundance, the abundance of each selected protein was z-score normalized across cells. The cell-cycle protein score for each cell was then calculated as the mean z-score across all detected cell-cycle-related proteins. ρ = Spearman correlation coefficient. Significance was considered at *p* < 0.05. Representative images of individual isolated cells in a piezo capillary dispenser are shown. PG, protein group; UMAP, uniform manifold approximation and projection.

The impact on cellular morphometry (diameter, elongation, and circularity data retrieved by cellenONE software; Data S1) was then explored. Overall, fresh cells presented higher median cell diameter and narrower elongation distribution than cryopreserved and fixed cells (Supplemental Table S6 and Fig. 3, *E* and *F*). While the cell circularity was narrower in fresh conditions when compared to fixed conditions, there was no significant variance with cryopreserved conditions (Supplemental Table S6 and Fig. 3*G*).

We further investigated the abundance of individual cell-cycle-related proteins to determine whether they contributed to the observed association between protein abundance and cellular volume. In total, 31 proteins related to the cell cycle were identified in our dataset, including cyclin-dependent kinases (CDK1, CDK2, CDK4, and CDK6) (35), DNA replication proteins (MCM family) (36), and mitotic and chromosome segregation proteins (PLK1, AURKA, AURKB, and CDC20) 37, 38, 39. The estimated cell volume was computed based on cell diameter and elongation data (see Experimental procedures), and a positive correlation with the cell-cycle protein score (See Experimental procedures) was observed in fresh cells and in several preservation conditions, including PFA 15 min for 1 day, PFA 30 min for 8 days, and cryopreservation at −80 °C for 1 day, whereas no significant associations were observed under the remaining conditions (Fig. 3*H*). Although this association did not reach statistical significance after 8 days of cryopreservation, the correlation remained slightly positive (ρ = 0.15), in contrast to the weak negative correlation observed in cells maintained at 4 °C for 1 day (ρ = −0.15). Representative images of isolated HeLa cells from each storage condition group are shown in Figure 3*E*.

The individual abundance of cell-cycle-related proteins and their contribution to the correlation with the cellular volume were also explored. Interestingly, some proteins showed positive correlations with cell volume across the evaluated conditions, including CDK2, AURKA, CCNB1, KIF11, BUB1B, MAD2L1, and CDC20, which are involved in cell-cycle progression, mitotic spindle organization, spindle assembly checkpoint activation, and the metaphase-to-anaphase transition 35, 38, 39. This pattern suggests that the observed association between cellular volume and the cell-cycle protein score may be driven predominantly by proteins associated with G2/M progression and mitotic regulation (40). In parallel, other replication-associated proteins showed weak or negative correlations, including MCM3, MCM5, and MCM7, which are components of the MCM2–7 replicative helicase complex involved in DNA replication initiation and progression through S phase (36). These results suggest that the relationship between cell volume and cell-cycle protein abundance is not homogeneous across cell-cycle processes, with proteins associated with G2/M phases and mitotic regulation showing stronger associations than proteins involved in DNA replication (Supplemental Fig. S2).

Additionally, we evaluated whether normalization according to cellular volume prior to UMAP analysis would alter the observed pattern. However, the same overall pattern was observed after normalization (Supplemental Fig. S3).

Finally, a total of 686 PGs, largely associated with metabolic processes, showed decreased abundance in fixed cells, whereas 35 PGs involved in biosynthetic pathways, such as cholesterol synthesis, showed increased abundance (Fig. 4 and Supplemental Table S7). Notably, cells incubated in PFA for 30 min had fewer abundant PGs than those incubated in PFA for 15 min, suggesting partial preservation of the proteome state. No significant differences were observed across any cryopreserved conditions (Fig. 4*A* and Supplemental Table S7). In contrast, cells stored at 4 °C for 1 day showed an increased abundance of PGs associated with pyroptosis (Fig. 4, *A* and *B*, and Supplemental Table S7), highlighting the importance of optimal cell storage conditions for subsequent proteomic analysis.

**Fig. 4:**
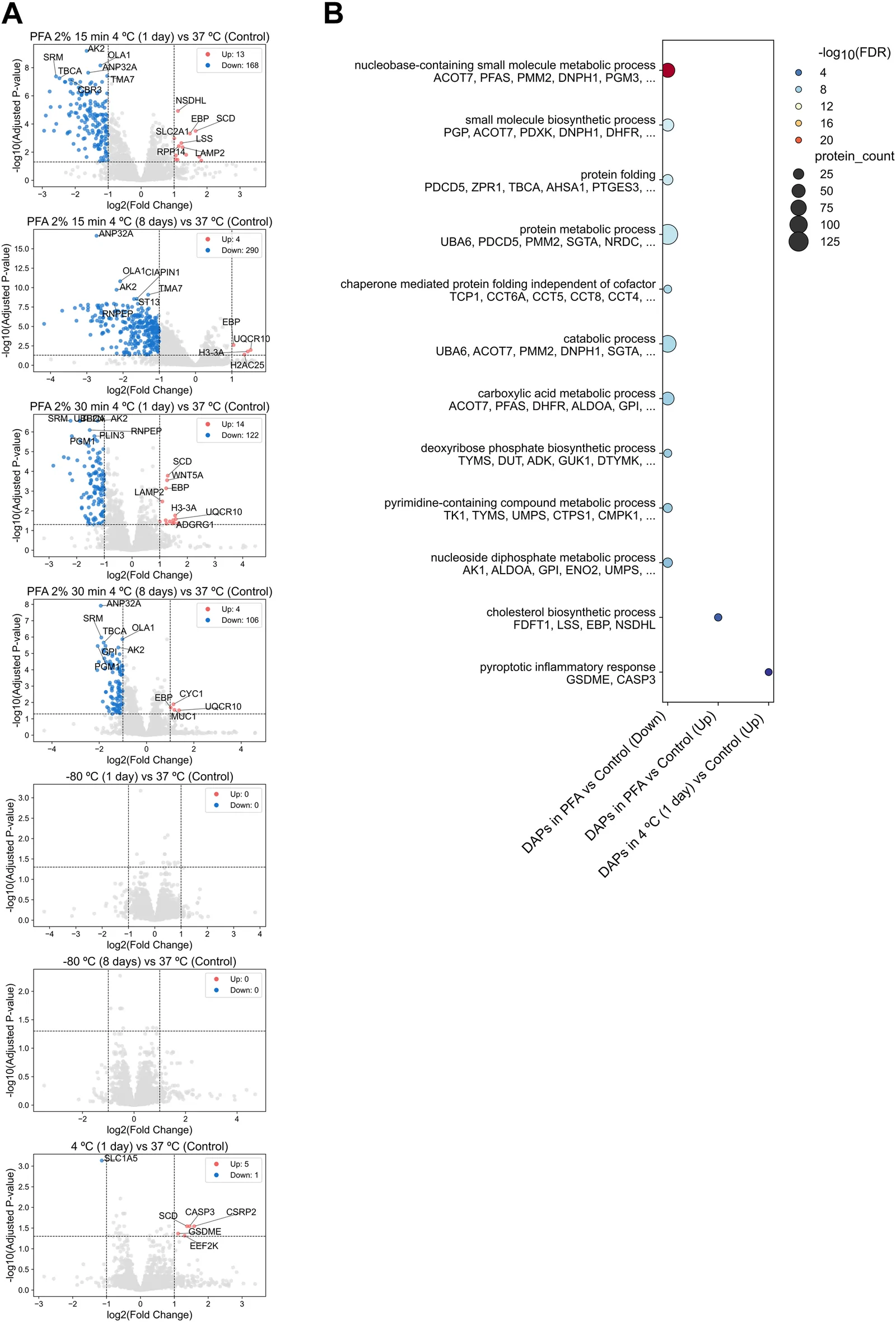
**Cryopreservation preserves the proteome of single HeLa cells, whereas fixation with paraformaldehyde induces proteomic changes.***A*, volcano plots of HeLa cells under different storage conditions *versus* control; log_2_(fold change) cutoffs were set to −1 and 1, and −log_10_ (adjusted *p*-value) cutoff to 1.3 (adjusted *p* < 0.05). *B*, Gene Ontology (biological processes) enrichment analysis of decreased (*down*) and increased (*up*) differentially abundant proteins in paraformaldehyde and 4 °C (1 day) conditions. *Dot color* indicates enrichment significance (adjusted *p* < 0.05, g:SCS correction), and *dot size* represents the number of overlapping proteins per term. Up to five representative gene symbols are displayed below each term.

Collectively, these results demonstrate that both fixation and cryopreservation are suitable preservation strategies for SCP, maintaining proteome coverage and variability after both short- and extended-term storage. Nevertheless, cryopreservation preserved proteomic profiles that were highly similar to those of freshly isolated cells. Although both preservation strategies affected cellular morphology (diameter, elongation, and circularity distributions), the association between estimated cell volume and the cell-cycle protein score was preserved across several preservation conditions. In summary, the choice of preservation strategy for downstream SCP analysis should consider the biological alterations associated with each approach.

### Cryopreservation was Compatible with SCP in PBMCs

Subsequently, we evaluated the effects of cryopreservation on PBMCs from a healthy donor to demonstrate the applicability of our SCP workflow to cryopreserved primary human immune cells.

On average, 750 PGs were identified in freshly collected and isolated PBMCs and 970 PGs in cryopreserved PBMCs for 8 days (Fig. 5*A* and Supplemental Table S8), with a maximum of 1499 PGs quantified in a single cryopreserved cell. Besides, although there were no significant differences among the CVs of fresh and cryopreserved (Fig. 5*B*), we observed an increase in the CV in PBMCs compared to HeLa cells, which may be due to the mixed cell populations present in blood and differences between *ex vivo* and *in vitro* conditions. A positive and significant correlation between the PG count and cell diameter was also observed (Fig. 5*C* and Supplemental Table S6). Although we did not observe significant variation of cell diameter, elongation, and circularity between fresh and cryopreserved cells (Fig. 5*C* and Supplemental Table S6), as well as cellular viability (approximately 3% cell death for both conditions) (Supplemental Fig. S4, *A–C*), cryopreserved cells showed a slight increased average of cell diameter (12.22 ± 1.23 μm, n = 75) compared with fresh cells (11.99 ± 1.61 μm, n = 77). This increase in cell diameter may have contributed to the higher average number of PGs identified in the cryopreserved condition, as larger cells may contain greater amounts of cellular material available for proteomic analysis (41).

**Fig. 5:**
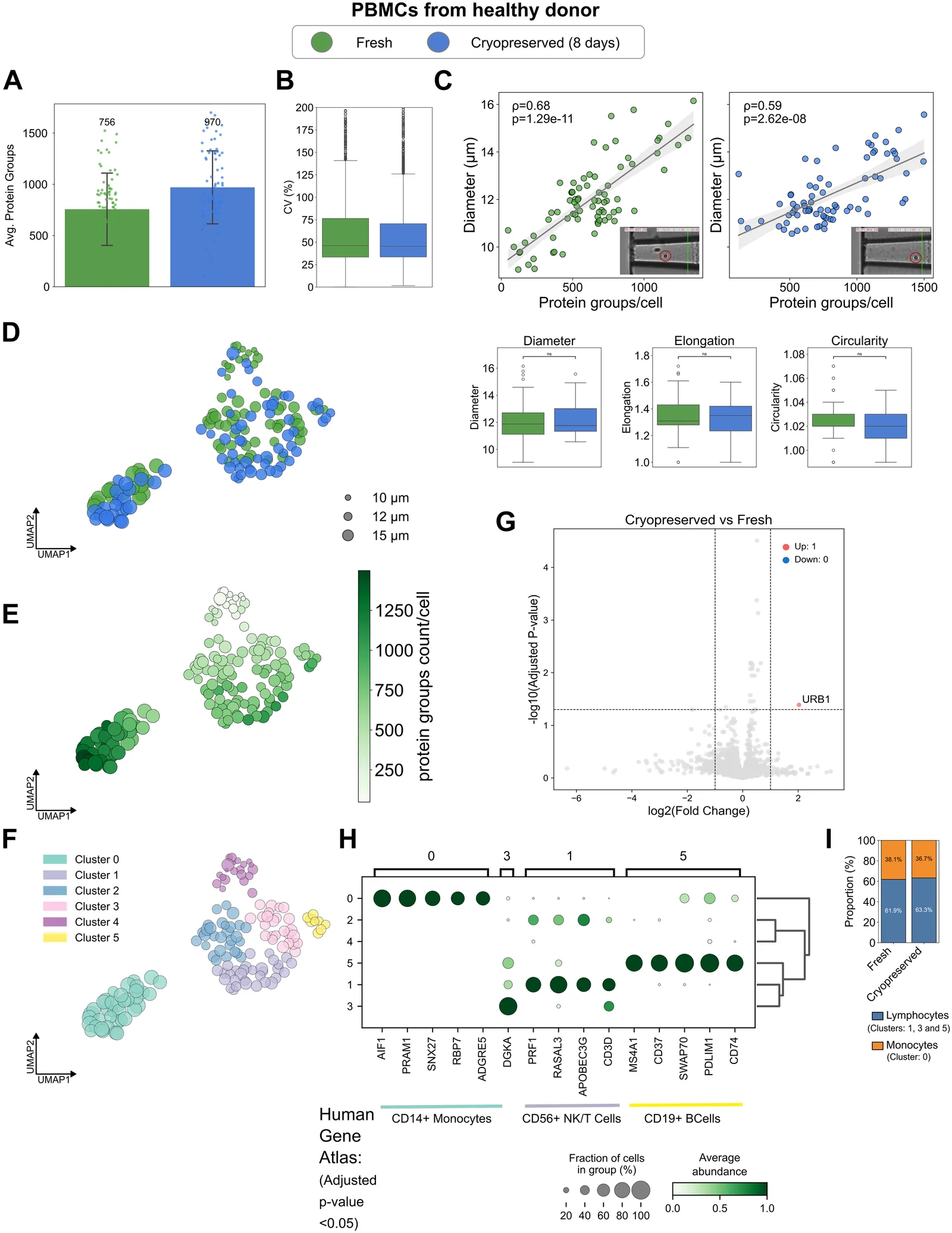
**Cryopreservation does not affect the proteome of single peripheral blood mononuclear cells from a healthy donor.***A*, average ± SD of PGs and (*B*) CV percentage of fresh (77 cells) and cryopreserved PBMCs (75 cells). *C*, correlation of cell diameter (μm) and PGs identified in PBMCs; ρ = Spearman correlation coefficient. Significance was considered at *p* < 0.05. Representative images of individual isolated cells in the piezo capillary dispenser are shown. *Boxplots* of single-cell diameter, elongation, and circularity distributions by PBMC conditions. Mann–Whitney U test with false discovery rate correction was used to determine the statistical differences between the groups; ns, not significant. *D* and *E*, UMAP plots colored according to the PBMC storage conditions (fresh and cryopreserved, 8 days) (*D*) and according to PG count per cell (*E*). The *dot size* in the UMAP plot corresponds to the cell diameter (range for isolated cells: 9–16 μm). *F*, volcano plot of differentially abundant proteins in fresh *versus* cryopreserved PBMCs. *Red dots* represent proteins with increased abundance in the cryopreserved cells (*Up*). Log_2_(fold change) cutoffs were set to −1 and 1, and −log_10_ (adjusted *p*-value) cutoff to 1.3 (adjusted *p* < 0.05). (*G*) UMAP colored according to the Leiden clusters (resolution of 1). *H*, *dot plot* showing the expression of canonical immune-cell marker proteins across the identified Leiden clusters. *Dot size* represents the percentage of cells expressing each protein within a cluster, and *color* intensity indicates the average normalized protein abundance. Cell-type identities were assigned based on canonical protein markers and supported by the Human Protein Atlas, identifying CD14^+^ monocytes (cluster 0), CD56^+^ NK/T cells (cluster 1), and CD19^+^ B cells (cluster 5). *I*, proportion of lymphocytes and monocytes identified in fresh and cryopreserved PBMCs based on single-cell proteomics clustering. PBMC, peripheral blood mononuclear cell; PG, protein group; UMAP, uniform manifold approximation and projection.

Importantly, to decrease background contamination during the experimental workflow, blank wells were added to the 384-well plates (n = 3 for fresh PBMCs; n = 2 for cryopreserved PBMCs). An average of 111 PGs was identified. The PGs found in both the blanks and PBMCs, and cells with fewer than 150 PGs, were excluded from the analysis (Supplemental Table S9). This cutoff was selected to retain cells with at least a 1.35-fold increase in the PG counts relative to the blank. PCA and UMAP projections revealed that fresh and cryopreserved PBMCs were grouped (Fig. 5, *D*–*F*, and Supplemental Table S10). Also, no proteins showed decreased abundance under cryopreserved conditions, and only a single protein (ribosome biogenesis factor URB1) exhibited increased abundance (Fig. 5*G* and Supplemental Table S11).

The analysis of the association between cellular volume and cell-cycle protein abundance, investigated in HeLa cells, is more challenging in PBMCs due to the variable nucleus-to-cytoplasm ratio across immune-cell subpopulations. Moreover, most circulating PBMCs, particularly naïve lymphocytes, are in a quiescent state and do not actively proliferate in peripheral blood. Upon antigen recognition and activation, lymphocytes undergo proliferation primarily within secondary lymphoid tissues, such as lymph nodes and the spleen (42). Based on these considerations, the UMAP analysis of PBMCs was based on proteome quantification for downstream identification of immune-cell subpopulations, which were subsequently correlated with cellular size.

Unsupervised Leiden clustering (resolution of 1) identified six proteomic clusters (Fig. 5*F*). Cell-type annotation based on canonical protein markers and the Human Gene Atlas (31) revealed clusters corresponding to major circulating immune-cell populations, including CD14^+^ monocytes (cluster 0), CD56^+^ NK/T cells (cluster 1), and CD19^+^ B cells (cluster 5) (Fig. 5*H* and Supplemental Table S12). Monocyte-enriched clusters were characterized by high abundance of AIF1, PRAM1, RBP7, and ADGRE5, whereas lymphoid populations expressed canonical markers such as PRF1, RASAL3, APOBEC3G, MS4A1, CD37, SWAP70, PDLIM1, and CD74. Importantly, cells from fresh and cryopreserved samples were distributed across all identified immune-cell populations, indicating that cryopreservation preserved the proteomic features required to identify major immune-cell populations in the analyzed PBMCs.

Finally, the proportion of cells classified as monocytes (cluster 0; CD14+ cells; Supplemental Fig. S5) and lymphocytes (clusters 1, 3, and 5; PTPRCAP+ cells; Supplemental Fig. S5) was assessed to evaluate whether cryopreservation affected the relative representation of these populations among the analyzed cells (Fig. 5*I*). In both conditions, lymphocytes accounted for a higher proportion of cells (61–63%) than monocytes (36–38%).

Overall, our results demonstrate that, under the conditions tested in this study, cryopreservation largely preserves the single-cell proteomic landscape of PBMCs without compromising cell-type resolution or quantitative proteome profiling. These findings support the feasibility of cryopreservation for SCP-compatible storage of primary immune-cell samples from healthy 43, 44 and disease donors (45), while recognizing that sample collection and preservation strategies may need to be adapted to the specific conditions, workflows, and resources available at different hospitals and laboratories.

## Discussion

The applicability of SCP approaches using clinical samples, such as those from blood and other liquid biopsies, is currently limited not only by mass spectrometry performance, but also by pre-analytical variables that remain poorly investigated (46). Unlike single-cell RNA sequencing, where RNA molecules are enzymatically amplified before sequencing, proteins cannot be amplified. Consequently, protein degradation, leakage, or extraction inefficiencies introduced during sample collection, preservation, transportation, or storage are irreversible and directly affect the depth and quality of the resulting proteome 17, 18, 19, 47, 48. Because SCP routinely measures only a fraction of the total cellular proteome, minimizing pre-analytical variability is especially important for robust biological interpretation. Establishing preservation strategies that maintain proteome integrity is, therefore, a critical step toward the routine implementation of SCP in clinical and translational research.

Our study addresses this challenge by first evaluating both fixation and cryopreservation strategies in single HeLa cells over both short- and extended-term storage. Notably, both preservation strategies worked well for downstream SCP analysis in terms of proteome coverage and variations. However, fixation significantly changed the proteomic landscape by decreasing the abundance of metabolic proteins. Based on these findings with HeLa cells, cryopreservation was selected as the preferred preservation strategy for subsequent clinical applications using PBMCs from one healthy donor. In summary, the effects of cryopreservation on the proteome were minimal even in heterogeneous immune-cell populations.

It is worth mentioning that the proteome depth observed in the single HeLa cells in this study (up to 3500 PGs per cell) was lower than that reported in some previous SCP studies (24). This difference may be partly explained by the sample preparation workflow used in the present study. The HeLa experiments used an earlier version of our sample preparation workflow 23, 24, whereas the protocol was subsequently optimized for the PBMC experiments. Removing the DMSO and trypsin enhancer from the lysis buffer for the digestion workflow resulted in improved PG identification in PBMCs, yielding proteome coverage comparable to that reported in previous SCP studies of PBMCs 44, 45, 49, 50 and neutrophils (10). Thus, comparisons of absolute proteome depth between HeLa and PBMC should not be made because the sample preparation workflows differed.

Overall, the choice of preservation strategy should be guided by several factors. The ability to fixate cells with PFA can be particularly attractive in studies involving clinical samples, as it allows samples to be stored for an extended period at 4 °C and is already a step widely used in flow cytometry workflows. By cross-linking proteins (21), PFA fixation can preserve samples during storage and facilitate sample handling and experimental scheduling. However, limited information is available on how long fixed samples can be stored without compromising proteome coverage, particularly at the single-cell level using clinical samples 18, 19. In our study, proteome coverage was not significantly affected by either short- or extended-term storage at 4 °C. However, despite maintaining comparable proteome coverage, all fixation conditions resulted in distinct proteomic profiles when compared to the freshly collected cells.

In parallel, cryopreservation can also be considered an attractive strategy because it does not introduce chemical modifications to the samples, thereby minimizing potential interference with proteome digestion and downstream proteomic analysis. In addition, this strategy is widely used in biobanks for the long-term storage of biological samples. Nevertheless, factors such as FBS proteins contamination, DMSO toxicity, modification of cell morphology, and changes in protein abundances stimulated by low temperature should be considered in SCP investigations. More recently developed freezing cell media free of both DMSO and FBS may help mitigate some of these limitations; however, their relatively high cost can limit their widespread application. In this study, we did not observe an increase in serum-derived proteins in cryopreserved samples compared with fixed and fresh cells. In addition, potential contaminants, including bovine proteins, were identified and excluded during data processing using a dedicated contaminant database.

In terms of logistics, cryopreservation requires maintenance of continuous freezing of the samples, typically involving dry ice or liquid nitrogen during sample storage and transportation, which may require dedicated infrastructure and periodic replenishment. In this study, we selected −80 °C cryopreservation rather than liquid nitrogen storage because it is directly compatible with dry-ice-based transportation and may facilitate sample shipment in laboratories without ready access to liquid nitrogen. This strategy may therefore be particularly practical for workflows requiring transfer of clinical samples between collection and analytical facilities. In contrast, fixation can reduce the dependence on cryogenic storage and facilitate transportation between laboratories, hospitals, and proteomics facilities, potentially allowing samples to be transported under refrigerated or, depending on the fixation and storage conditions, ambient conditions. Nevertheless, fixation does not eliminate the need for appropriate temperature and storage control, as prolonged exposure to suboptimal conditions may affect protein integrity. Thus, the choice between cryopreservation and fixation should consider the logistical requirements and infrastructure available for the study, as well as the intended downstream applications and whether additional analyses involving the cells are planned before or after SCP analysis.

Although our study focused on suspended cells, preservation strategies may have different implications for SCP workflows applied to tissue biopsies. For instance, formalin-fixed paraffin-embedded frozen tissues are commonly stored as tissue sections on glass slides, from which individual cells cannot be directly isolated as in suspended-cell workflows using the cellenONE system. Instead, additional steps are required to identify and isolate individual cells or regions of interest, such as laser microdissection. After laser microdissection, the cellenONE would be suitable for SCP sample preparation 46, 51. This approach has been further developed through single-cell deep visual proteomics, which combines high-resolution imaging with laser microdissection to enable the selection and untargeted proteomic analysis of individual cell shapes from tissue sections 52, 53. Therefore, the preservation strategies evaluated here for suspended cells should not necessarily be directly extrapolated to tissue-based single-cell proteomics workflows, in which tissue processing, spatial context, and cell recovery introduce additional preanalytical considerations.

### Limitations of the Study

This study was designed to systematically evaluate different preservation strategies using fixation and cryopreservation methods to facilitate future clinical SCP workflows using liquid biopsy samples. However, some limitations should be acknowledged. Extended storage periods (months or even years) representative of long-term biobanking should be investigated to further establish the robustness of these preservation strategies for large-scale clinical studies. In addition, the reduction in the number of proteins related to metabolic processes observed across all PFA conditions warrants further investigation, including experiments aimed at assessing the potential impact of crosslinking reversal, representing a challenging step in SCP experiments.

Moreover, the applicability of the workflow to primary human samples was evaluated using PBMCs from a single healthy donor. While this design was appropriate for validating the technical performance of the preservation strategy, rather than characterizing biological variability, future studies including larger and more diverse donor cohorts will be important to confirm the general applicability of these findings across different individuals and clinical conditions (45).

Finally, we envision that this study will provide an important description for researchers seeking to implement SCP workflows using biological and clinical samples, especially those from liquid biopsies 43, 44, 45, 46, 54, offering practical guidance for selecting preservation strategies according to sample handling, transportation, storage requirements, and experimental objectives.

## Data Availability

All data are available in the main text or supplementary materials. The mass spectrometry raw files and cellenONE reports were deposited at PRIDE under the code access: PXD071894. The code generated and files related to this work are publicly available in a GitHub repository (https://github.com/HeloisaMonteiroAP/SCP_Paper_Storage) and Zenodo repository (DOI: 10.5281/zenodo.22057663).

## Supplemental Data

This article contains supplemental data.

## Competing Interest

The authors declare they have no competing interests.
