# Supplementary material for: Benchmarking Sample Preservation Strategies for Clinical Single-Cell Proteomics
Source: Mol Cell Proteomics. 2026 Oct 6;25(11):101676. doi: 10.1016/j.mcpro.2026.101676 (PMC13756094; doi:10.1016/j.mcpro.2026.101676)
Supplement: Supplementary file 1 [file mmc1.docx]

**Supplementary Materials**

**Benchmarking sample preservation strategies for clinical single-cell proteomics**

Heloísa Monteiro do Amaral-Prado^1^, Jackson Gabriel Miyamoto^1^, Daniella de Figueiredo^1^, Elaine Cristina Cardoso¹, Fábio Malta de Sá Patroni^1^, Romênia Ramos Domingues^1^, Bianca Alves Pauletti^1^, Adriana Franco Paes Leme^1^*

**Affiliations:**

^1^Laboratório Nacional de Biociências (LNBio), Centro Nacional de Pesquisa em Energia e Materiais (CNPEM), Campinas, SP, 13083-970, Brazil;

*Corresponding author. Email: [adriana.paesleme@lnbio.cnpem.br](mailto:adriana.paesleme@lnbio.cnpem.br)

# Figure S1. Cell viability analysis of HeLa cells under different storage conditions. HeLa cells were subjected to the indicated storage conditions: fresh at 37 °C (control), 4 °C for 1 or 8 days, 2% PFA fixation for 15 or 30 min followed by storage at 4 °C for 1 or 8 days, and −80 °C for 1 or 8 days. After storage, cells were stained with Zombie dye (APC-Cy7). The cellenONE system, equipped with a red LED, was used for fluorescence detection. Cells positive for red fluorescence (Zombie APC-Cy7) were considered non-viable. Scatter plots show individual cells (n = 100 cells analyzed per condition), with cell diameter (µm) plotted against fluorescence intensity. Red dots represent Zombie-positive (non-viable) cells. The percentage of marked (non-viable) cells was automatically calculated by the cellenONE system and is displayed for each condition.

**Figure S2. Heatmap of Spearman correlations between the abundance of cell-cycle-related proteins and cell volume of single HeLa cells under different storage conditions.**

Positive correlations indicate higher protein abundance in larger cells, whereas negative correlations indicate higher protein abundance in smaller cells. Correlation coefficients are shown within each storage condition, with color intensity representing the strength and direction of the correlation.

**Figure S3. UMAP visualization before and after normalization according to single HeLa cells volume (µm³).**

Each dot represents an individual cell, with dot size according to the estimated cell volume calculated based on the diameter and elongation parameters retrieved by cellenONE software. In the original embedding, protein abundances were log-transformed and scaled before PCA and UMAP analysis. For the volume-adjusted embedding, the effect of estimated cell volume was regressed out from the log-transformed protein abundance matrix before scaling, PCA, and UMAP construction. Colors indicate experimental conditions, while the color gradient in the lower panels represents the number of protein groups quantified per cell. This analysis was performed to evaluate whether the observed proteomic heterogeneity was influenced by differences in cell size.

**Figure S4. Cell viability analysis of fresh and cryopreserved PBMCs.**

(**A**) Representative detection of Zombie NIR™ fluorescence in fresh and cryopreserved PBMC suspensions using the cellenONE X1 imaging system. The cellenONE system, equipped with a red LED, was used for fluorescence detection. Cells positive for red fluorescence (Zombie APC-Cy7) were considered non-viable. Scatter plots show individual cells (n = 100 for fresh cells and n = 300 for cryopreserved cells), with cell diameter (µm) plotted against fluorescence intensity. Red dots represent Zombie-positive (non-viable) cells. The percentage of marked (non-viable) cells was automatically calculated by the cellenONE system and is displayed for each condition. (**B**) Representative dead cell showing positive Zombie NIR™ fluorescence (magenta circle). (**C**) Representative viable PBMC lacking Zombie NIR™ fluorescence (green circle).

**Figure S5. UMAPs of PBMCs colored according to the protein abundance of CD14 (monocyte marker) and PTPRCAP (lymphocyte marker).**

Each point represents an individual cell, with darker green indicating higher protein abundance.

Data S1. Explanation of morphometric parameters retrieved by cellenONE X1 software.

**Diameter:** This parameter represents the diameter of a disk with the same area as the particle; Δ = 2√(S/π).

**Elongation:** This parameter is used to differentiate two aggregated cells from a single cell, as these particles will have different elongation factors. The more elongated the shape of a particle, the higher its elongation factor. The elongation factor is measured with the following equation: max Feret diameter/[equivalent rect. (short side) (Feret)];

ε = D/Rf.

**Circularity:** This parameter measures how closely a cell's shape resembles a perfect circle, calculated as a ratio, where 1.0 represents a perfect circle and values closer to 0 indicate more elongated or irregular shapes.

Data S2. Spectronaut parameters.

|  | **Spectronaut v19.9** | **Spectronaut v20.1** |
| --- | --- | --- |
| **Pulsar Search** | | |
| Enzyme/cleavage rules | Trypsin/P | Trypsin/P |
| Digestion type | Specific | Specific |
| Decoy generation rule | KR | KR |
| Maximum peptide length | 52 | 52 |
| Minimum peptide length | 7 | 7 |
| Missed cleavages | 2 | 2 |
| Toggle N-terminal M excision | True | True |
| Search mode |  | Closed search |
| Max variable modifications | 5 | 5 |
| Variable modifications | Acetyl (protein N-term) and oxidation (M) | Acetyl (protein N-term) and oxidation (M) |
| Grouped peptide FDR |  | None |
| PSM FDR | 0.01 | 0.01 |
| Peptide FDR | 0.01 | 0.01 |
| Protein group FDR | 0.01 | 0.01 |
| DirectDIA workflow | DirectDIA+ (Deep) | DirectDIA+ (Deep) |
| PTM localization filter | False | False |
| Tolerance parameter |  |  |
| Calibration search | Dynamic | Dynamic |
| MS1 correction factor | 1 | 1 |
| MS2 correction factor | 1 | 1 |
| **DIA Analysis** | | |
| Precursor *q* value cutoff | 0.01 | 0.01 |
| Precursor *q* value cutoff (experiment) | NA | 0.01 |
| Precursor PEP cutoff | 0.2 | 0.2 |
| Protein *q* value cutoff (experiment) | 0.01 | 0.01 |
| Protein *q* value cutoff (run) | 0.05 | 0.05 |
| Protein PEP cutoff | 0.75 | 0.75 |
| Single hit definition | By stripped sequence | By stripped sequence |
| Single hit protein rule | NA | Stratified single hit protein FDR |
| Run-level protein scoring | NA | All observations |
| Exclude single hit proteins | Off | NA |
| Pvalue estimator | Kernel density estimator | Kernel density estimator |
| Precursor filtering | Identified (*q* value) | Identified (*q* value) |
| Proteotypicity filter | None | None |
| Protein LFQ method | Automatic | Automatic |
| Quantity MS1 level | MS1 | MS1 |
| Quantity type | Area | Area |
| Cross-run normalization | On | On |
| Inference correction | On | On |
| Major (protein) grouping | By protein group ID | By protein group ID |
| Minor (peptide) grouping | By stripped sequence | By stripped sequence |
| Major group quantity | Mean peptide quantity | Mean peptide quantity |
| Major group top N | On | On |
| Minor group quantity | Mean precursor quantity | Mean precursor quantity |
| Minor group top N | On | On |

Data S3. DIA-NN parameters.

|  | **DIA-NN v2.3** |
| --- | --- |
| Mode | Library search/off |
| Protease | Trypsin/P |
| Missed cleavages | 2 |
| Mass variable modifications | 1 |
| Contaminants | On |
| N-term M | On |
| Acetyl (N-term) | On |
| Peptide length range | 7–30 |
| Precursor charge range | 1–4 |
| Precursor m/z range | 300–1800 |
| Fragment ion m/z range | 200–1800 |
| FDR (%) | 1 |
| MS1 accuracy | 4 |
| MS2 accuracy | 10 |
| MBR | On |
| Protein inference | On |
| Scoring | Peptidoforms |
| Proteotypicity | Genes |
| Machine learning | NNs (cross-validated) |
| Quantification strategy | QuantUMS (precision) |
| Cross-run normalization | RT-dependent |
| Library generation | IDs, RT and IM profiling |
| Speed peak filtering | Optimal results |
| Speed RT/IM filtering | Balanced |
